# Paternal genetic variants and risk of obstructive heart defects: A parent-of-origin approach

**DOI:** 10.1371/journal.pgen.1009413

**Published:** 2021-03-08

**Authors:** Jenil Patel, Emine Bircan, Xinyu Tang, Mohammed Orloff, Charlotte A. Hobbs, Marilyn L. Browne, Lorenzo D. Botto, Richard H. Finnell, Mary M. Jenkins, Andrew Olshan, Paul A. Romitti, Gary M. Shaw, Martha M. Werler, Jingyun Li, Wendy N. Nembhard, the National Birth Defects Prevention Study

**Affiliations:** 1 Department of Epidemiology, Fay W. Boozman College of Public Health, University of Arkansas for Medical Sciences, Little Rock, AR, United States of America; 2 Arkansas Center for Birth Defects Research and Prevention, Fay W. Boozman College of Public Health, University of Arkansas for Medical Sciences, Little Rock, AR, United States of America; 3 Department of Epidemiology, Human Genetics and Environmental Sciences, The University of Texas Health Science Center at Houston (UTHealth) School of Public Health, Dallas, TX, United States of America; 4 Biostatistics Program, Department of Pediatrics, College of Medicine, University of Arkansas for Medical Sciences, Arkansas Children’s Research Institute, Little Rock, AR, United States of America; 5 Rady Children’s Institute for Genomic Medicine, San Diego, CA, United States of America; 6 Birth Defects Research Section, New York State Department of Health, Albany, NY, United States of America; 7 Department of Epidemiology and Biostatistics, School of Public Health, University at Albany, Rensselaer, NY, United States of America; 8 Division of Medical Genetics, Department of Pediatrics, University of Utah, Salt Lake City, UT, United States of America; 9 Department of Molecular and Cellular Biology, Center for Precision Environmental Health, Baylor College of Medicine, Houston, TX, United States of America; 10 National Center on Birth Defects and Developmental Disabilities, Centers for Disease Control and Prevention, Atlanta, GA, United States of America; 11 Department of Epidemiology, Gillings School of Global Public Health, University of North Carolina at Chapel Hill, Chapel Hill, NC, United States of America; 12 Department of Epidemiology, College of Public Health, The University of Iowa, Iowa City, IA, United States of America; 13 Division of Neonatal and Developmental Medicine, Department of Pediatrics, Stanford University School of Medicine, Stanford, CA, United States of America; 14 Department of Epidemiology, School of Public Health, Boston University, Boston, MA, United States of America; Indiana University at Bloomington, UNITED STATES

## Abstract

Previous research on risk factors for obstructive heart defects (OHDs) focused on maternal and infant genetic variants, prenatal environmental exposures, and their potential interaction effects. Less is known about the role of paternal genetic variants or environmental exposures and risk of OHDs. We examined parent-of-origin effects in transmission of alleles in the folate, homocysteine, or transsulfuration pathway genes on OHD occurrence in offspring. We used data on 569 families of liveborn infants with OHDs born between October 1997 and August 2008 from the National Birth Defects Prevention Study to conduct a family-based case-only study. Maternal, paternal, and infant DNA were genotyped using an Illumina Golden Gate custom single nucleotide polymorphism (SNP) panel. Relative risks (RR), 95% confidence interval (CI), and likelihood ratio tests from log-linear models were used to estimate the parent-of-origin effect of 877 SNPs in 60 candidate genes in the folate, homocysteine, and transsulfuration pathways on the risk of OHDs. Bonferroni correction was applied for multiple testing. We identified 3 SNPs in the transsulfuration pathway and 1 SNP in the folate pathway that were statistically significant after Bonferroni correction. Among infants who inherited paternally-derived copies of the G allele for rs6812588 in the *RFC1* gene, the G allele for rs1762430 in the *MGMT* gene, and the A allele for rs9296695 and rs4712023 in the *GSTA3* gene, RRs for OHD were 0.11 (95% CI: 0.04, 0.29, P = 9.16x10^-7^), 0.30 (95% CI: 0.17, 0.53, P = 9.80x10^-6^), 0.34 (95% CI: 0.20, 0.57, P = 2.28x10^-5^), and 0.34 (95% CI: 0.20, 0.58, P = 3.77x10^-5^), respectively, compared to infants who inherited maternally-derived copies of the same alleles. We observed statistically significant decreased risk of OHDs among infants who inherited paternal gene variants involved in folate and transsulfuration pathways.

## Author summary

Obstructive heart defects are birth defects that cause obstruction to the blood flow of the developing heart. Common OHDs include coarctation of the aorta, aortic stenosis and pulmonary stenosis. While there is a fair amount of literature indicating an association between maternal genetic variants and OHDs, less is known about the role of paternal genetic variants in the etiology of OHDs. We used a genotype clustering algorithm, SNPMClust, that was developed in-house at the Arkansas Center for Birth Defects Research and Prevention to study the role of paternal genetic variants in the folate, homocysteine and transsulfuration pathways. Maternal, paternal, and infant DNA specimens were collected from participants of the National Birth Defects Prevention Study, a large population-based case-control study in the United States, and were genotyped using an Illumina Golden Gate custom single nucleotide polymorphism (SNP) panel. We identified 4 SNPs in the folate and transsulfuration pathways, rs6812588, rs1762430, rs9296695, and rs4712023, that were associated with a statistically significant decreased risk of OHDs for infants who inherited a paternally-derived copy of the variant allele compared to infants who inherited a maternal copy of the variant allele. In conclusion, we observed a significantly decreased risk and less epigenetic influence of paternal genetic variants on OHDs compared to maternally-derived variants.

## Introduction

Congenital heart defects (CHDs) are the most commonly occurring group of birth defects and affect about one percent of live births in the United States annually [1, 2]. They are also the most common cause of infant mortality and lifelong morbidity [3, 4]. It has also been reported that overall survival among patients with complex heart defects is decreased with increasing age compared to healthy age-matched counterparts [3]. Although some CHDs occur in association with certain genetic syndromes (e.g., trisomy 21, 22q11 deletion, Alagille syndrome, Noonan syndrome) and teratogenic exposures (e.g., anticonvulsants, maternal pregestational diabetes), approximately 80% are of unknown etiology [5–7]. Known maternal risk factors associated with CHDs include diabetes mellitus [8], obesity [9], prenatal cigarette smoking [10–15], low blood folate concentrations [16], hyperhomocysteinemia [17], medication use [18] and genetic polymorphisms in metabolic pathways, including the folate, homocysteine, and glutathione/transsulfuration pathways [8, 9, 13–33].

In contrast, the role of paternal environmental and genetic factors on the risk of CHDs is less defined with limited literature [34]. Some studies indicate associations between young or advanced paternal age and increased risk of atrial septal defects, ventricular septal defects, right ventricular outflow tract defects including pulmonary valve atresia, patent ductus arteriosus, and CHDs overall [35, 36]; however, other studies report no association between paternal age and risk of CHDs. Other paternal exposures associated with increased risk of CHDs include cigarette smoking, alcohol consumption, and occupational exposure to endocrine disruptors [11, 35–43].

Although the specific biological mechanisms are unclear, it is hypothesized that these exposures (paternal age, smoking, etc.) may share a similar physiologic mechanism: germline mutations and epigenetic alterations to sperm haploid DNA [10, 44–49]. Given that environmental exposures may induce changes in paternal DNA that can result in CHDs, we postulated that certain paternal genetic polymorphisms may also increase CHD risk. Numerous studies confirm that genetic polymorphisms in maternal and infant genes are directly or indirectly associated with risk of CHDs, particularly genes involved in folate, homocysteine, and transsulfuration pathways [19, 20, 50].

To our knowledge, to date (as of March 2020), only one study has assessed the influence of paternal genetic variants in folate, homocysteine, or transsulfuration pathways and CHD risk in offspring [51]. This study examined conotruncal heart defects and found less epigenetic influence on conotruncal heart defects by paternal genetic variants compared to maternal genes. No studies have assessed other groups of CHDs, such as right-sided and left-sided obstructive heart defects (OHDs). Among subtypes of OHDs, pulmonary stenosis and coarctation of the aorta account for 8% and 5% of all CHDs respectively [2], making OHDs an important group of CHDs to investigate. Moreover, studies show that women who delivered infants with OHDs were more likely to have alterations in metabolites in pathways involving folate, homocysteine, and glutathione [25, 52, 53]. Additionally, a recent study demonstrated that risk of OHDs was closely related to a combined effect of variations in genes in the folate, homocysteine, or glutathione/transsulfuration pathways, maternal use of folic acid supplements and pre-pregnancy obesity, although the focus was on genetic variants in maternal genes [54]. Whether a similar pattern of gene-environment interaction effects for paternal exposures, including alterations in pathways for paternal genes, are also responsible for causing OHDs is yet to be explored. In this study, we investigated parent-of-origin effects for genetic variants in folate, homocysteine and transsulfuration pathway genes and the occurrence of OHDs in offspring.

## Results

Table 1 displays the distributions of maternal and paternal characteristics of infants born with OHDs for whom genotyping was performed. The mean maternal and paternal age at delivery were 28.3 (6.0) and 32.0 (6.7), respectively. As for race/ethnicity, 73.2% and 67.9% of mothers and fathers, respectively, were non-Hispanic white. Approximately 30% and 26% of mothers and fathers, respectively, had some college education. Among mothers, 26.1% were overweight, and 23.0% were obese. About 58% of the mothers took folic acid supplements during the periconceptional period (a month before conception through the end of the first trimester); 20.4% drank some quantity of alcohol during the entire pregnancy (date of conception through date of birth) and 13.5% smoked cigarettes during the entire pregnancy period. Among fathers, 94% were employed at the time of interview, while 18% had a birth defect or health problem at birth.

**Table 1:** Summary of maternal and paternal characteristics from chi-squared analyses for mothers of infants with obstructive heart defects, The National Birth Defects Prevention Study, USA, October 1997 –August 2008 births (n = 569 case families)^a^.

Characteristics	Maternal	Paternal
	n (%)	n (%)
Age at Delivery		
Mean (SD)	28.3 (6.0)	32.0 (6.7)
<35 years	480 (84.4%)	285 (71.6%)
≥35 years	89 (15.6%)	113 (28.4%)
Missing	0	171
**Race/Ethnicity**		
Non-Hispanic white	413 (73.2)	383 (67.9)
Non-Hispanic black	61 (10.8)	69 (12.2)
Hispanic	66 (11.7)	77 (13.7)
Other	24 (4.3)	25 (4.4)
Missing	0	10
**Education**		
< 12 years	73 (12.9)	81 (14.4)
High school diploma or equivalent	139 (24.6)	155 (27.5)
< 4 years of college education	170 (30.1)	144 (25.5)
At least 4 years of college or bachelor’s degree	182 (32.2)	171 (30.3)
Missing	0	13
**Mean Household Income**		
< $10,000	70 (12.4)	N/A
$10,000 –$29,999	163 (28.9)	N/A
$30,000 –$49,999	129 (22.9)	N/A
≥ $50,000	180 (31.9)	N/A
Missing	22	N/A
**Body Mass Index**		
Underweight (< 18.5 kg/m^2^)	14 (2.5)	N/A
Normal weight (18.5 to < 25.0 kg/m^2^)	257 (45.6)	N/A
Overweight (25.0 to <30.0 kg/m^2^)	147 (26.1)	N/A
Obese (≥ 30.0 kg/m^2^)	130 (23.0)	N/A
Missing	16	N/A
**Periconceptional Folic Acid Supplementation**		
No	236 (41.8)	N/A
Yes	328 (58.2)	N/A
**Alcohol Intake**^**b**^		
No	445 (78.9)	N/A
Yes	115 (20.4)	N/A
Missing	4	N/A
**Cigarette Smoking**^**b**^		
No	488 (86.5)	N/A
Yes	76 (13.5)	N/A
**Cigarette Smoking in Home During First Trimester**		
No	471 (83.5)	N/A
Yes	93 (16.5)	N/A
**Currently Employed (at Time of Interview)**		
No	N/A	28 (5.0)
Yes	N/A	532 (94.3)
Missing information	N/A	4
**Health Problem at Birth or a Birth Defect Diagnosed in Childhood?**		
No	N/A	452 (80.1)
Yes	N/A	100 (17.7)
Missing information	N/A	12
**Mother Blood Relative of Baby’s Father?**		
No	N/A	559 (99.1)
Yes	N/A	3 (0.9)
Missing information	N/A	2

a. Data are from families for whom DNA samples were available

b. During pregnancy = date of conception to date of birth

N/A = Not Available

The final analysis included 877 SNPs within 60 genes. Based on Bonferroni adjustment, the statistical significance was set at ≤ 5.70×10^−5^. We observed a statistically significant *decreased* risk of OHDs for paternally-derived effects for four SNPs in three genes (Table 2): one SNP each in replication factor C subunit1 (*RFC1*) and O-6-methylguanine-DNA methyltransferase (*MGMT*); and two SNPs in glutathione S-transferase alpha 3 (*GSTA3*). These genes are involved in DNA replication and repair, catalyzing transfer of methyl groups, and cellular defense.

**Table 2:** Risk ratios and 95% confidence intervals (CIs) with p-values for paternally-derived effects for the top 20 single nucleotide polymorphisms (SNPs) identified from hybrid analyses compared to maternally-derived effects for common variants in genes involved in folate, homocysteine and transsulfuration pathways and risk of obstructive heart defects, The National Birth Defects Prevention Study, USA, October 1997 –August 2008 births (n = 569 case families).

SNP	Referent/Risk allele	Chr	Gene	Pathway	Relative Risk (95% CI)	P-value^a^
rs6812588	**G/A**	**4**	***RFC1***	**Folate**	**0.11 (0.04, 0.29)**	**9.16×10**^**−7**^
rs1762430	**G/A**	**10**	***MGMT***	**Transsulfuration**	**0.30 (0.17, 0.53)**	**9.80×10**^**−6**^
rs9296695	**A/G**	**6**	***GSTA3***	**Transsulfuration**	**0.34 (0.20, 0.57)**	**2.28×10**^**−5**^
rs4712023	**A/G**	**6**	***GSTA3***	**Transsulfuration**	**0.34 (0.20, 0.58)**	**3.77×10**^**−5**^
rs9299871	A/G	10	*MGMT*	Transsulfuration	0.22 (0.10, 0.49)	8.81×10^−5^
rs7541539	A/C	1	*MTR*	Homocysteine	0.27 (0.14, 0.53)	8.81×10^−5^
rs7069462	A/G	10	*MGMT*	Transsulfuration	0.20 (0.09, 0.47)	1.02×10^−4^
rs2273027	A/G	17	*SHMT1*	Folate	2.14 (1.46, 3.13)	1.27×10^−4^
rs12202200	A/G	6	*GSTA3*	Transsulfuration	0.36 (0.20, 0.64)	2.79×10^−4^
rs6577	C/A	6	*GSTA2*	Transsulfuration	0.31 (0.16, 0.60)	2.90×10^−4^
rs7818511	A/G	8	*GSR*	Transsulfuration	0.38 (0.22, 0.65)	2.93×10^−4^
rs600473	C/A	5	*BHMT*	Homocysteine	1.87 (1.34, 2.62)	3.19×10^−4^
rs9382157	A/G	6	*GSTA3*	Transsulfuration	0.37 (0.21, 0.65)	4.18×10^−4^
rs1547177	A/C	10	*MGMT*	Transsulfuration	0.35 (0.19, 0.65)	5.67×10^−4^
rs2152151	C/G	10	*MGMT*	Transsulfuration	0.34 (0.18, 0.66)	7.28×10^−4^
rs2062228	A/G	4	*RFC1*	Folate	0.24 (0.10, 0.56)	1.11×10^−3^
rs2424905	A/G	20	*DNMT3B*	Homocysteine	0.55 (0.38, 0.80)	1.31×10^−3^
rs2363641	G/A	14	*GSTZ1*	Transsulfuration	1.90 (1.28, 2.80)	1.46×10^−3^
rs4796017	G/A	17	*NOS2A*	Transsulfuration	1.75 (1.23, 2.50)	2.53×10^−3^
rs7081756	C/A	10	*MAT1A*	Homocysteine	0.54 (0.36, 0.82)	2.54×10^−3^

a. “Bolded p-values are for significant SNPs with p-value ≤ 5.70x10^-5^.”

Among infants who inherited a paternally-derived copy of the G allele for rs6812588 in the *RFC1* gene, the relative risk (RR) of an OHD was 0.11 (95% confidence interval [CI]: 0.04, 0.29, *p* = 9.16x10^-7^) compared to infants who inherited a maternal G allele. In the *MGMT* gene, for infants who inherited a paternally-derived copy of the G allele for rs1762430, the RR was 0.30 (95% CI: 0.17, 0.53, *p* = 9.80x10^-6^) compared to infants who inherited the maternal G allele. Among infants who inherited a paternally-derived copy of the A allele for rs9296695 or rs4712023 in *GSTA3*, the RRs were 0.34 (95% CI: 0.20, 0.57, *p* = 2.28x10^-5^) and 0.34 (95%CI: 0.20, 0.58, *p* = 3.77x10^-5^), respectively, compared to infants who inherited the maternally-derived A allele.

Elevated, non-significant risks were observed for infants who inherited paternally-derived copies of the: (i) A allele for rs2273027 in the *SHMT1* gene in the folate pathway (RR 2.14, p = 1.27×10^−4^); (ii) C allele for rs600473 in the *BHMT* gene in the homocysteine pathway (RR 1.87, p = 3.19x10^-4^); and (iii) G allele for rs2362641 and rs4796017 in the *GSTZ1* and *NOS2A* genes, respectively in the transsulfuration pathway (RR 1.90, p = 1.46x10^-3^ and RR 1.75, p = 2.53x10^-3^, respectively), compared to infants who inherited maternally-derived copies.

**Fig 1:**
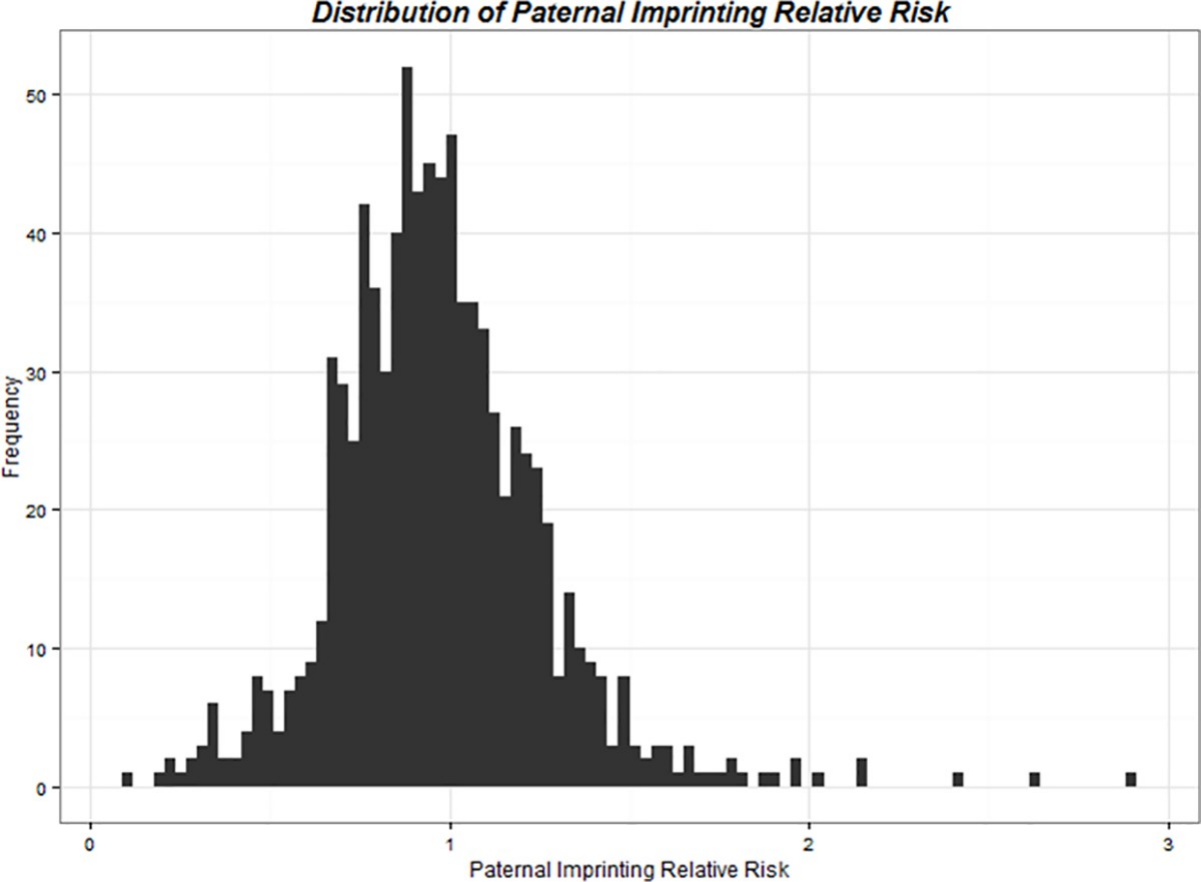
Distribution of relative risks, identified from hybrid analyses, for obstructive heart defects in infants with paternally-derived compared to maternally-derived risk variants in genes involved in folate, homocysteine, and transsulfuration pathways, The National Birth Defects Prevention Study, USA, October 1997–August 2008 births.

**Fig 2:**
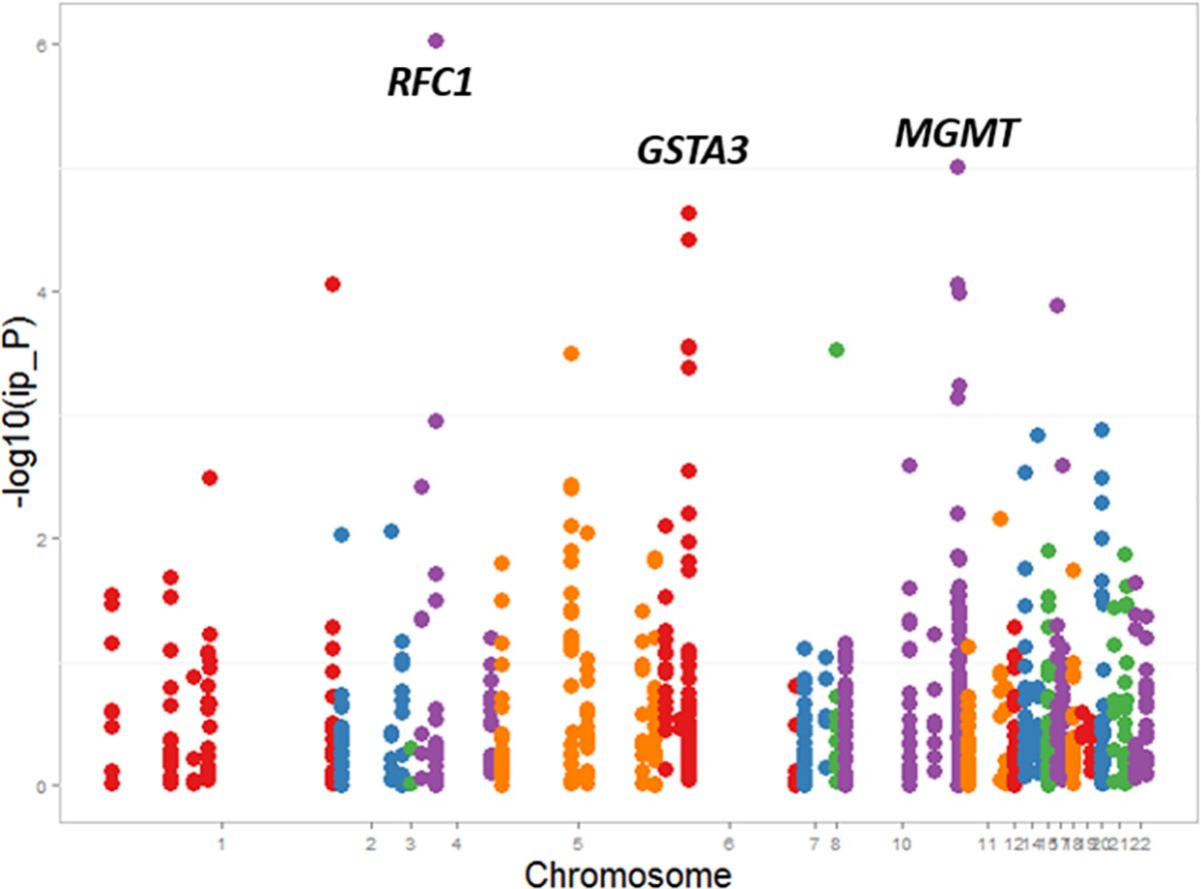
Manhattan plot that shows the location of genes involved in folate, homocysteine, and transsulfuration pathways for which we observed, from hybrid analyses, a significantly decreased risk of obstructive heart defects when the risk variant was paternally-derived compared to maternally-derived, The National Birth Defects Prevention Study, USA, October 1997–August 2008 births.

Fig 1 shows the distribution of RRs, identified from hybrid analyses, for OHDs in infants who inherited a paternally-derived risk variant compared to a maternally-derived risk variant in genes involved in all three pathways. A Manhattan plot was also constructed (Fig 2) to display the location of specific genes involved in all three pathways for which we observed, from hybrid analyses, a significantly decreased risk of OHDs when the risk variant was paternally-derived compared to maternally-derived.

In summary, we observed a statistically significant decreased risk of OHDs among infants who inherited a paternally-derived copy of one folate and three transsulfuration pathway genes compared to maternally-derived variants.

## Discussion

In this study, we determined the parent-of-origin effects for genetic variants in folate, homocysteine, and transsulfuration pathway genes and occurrence of OHDs in offspring. The majority of published studies have investigated the effects of maternal genetic variants and environmental factors on the occurrence of CHDs in general [12, 55–57]; however, much less is known about the role of paternally-related genetic variants in outcomes of pregnancy, including their roles in CHD etiology [51]. We observed no statistically significant increased risk of OHDs for infants who inherited a paternally-derived copy of variant alleles in genes involved in folate, homocysteine, or transsulfuration pathways compared to infants who inherited a maternal copy of the variant allele. However, we did identify 4 SNPs (rs6812588, rs1762430, rs9296695, and rs4712023) that were associated with a statistically significant decreased risk of OHDs for infants who inherited a paternally-derived copy compared to infants who inherited a maternal copy of the variant allele (Fig 2).

One possible explanation for our results, as suggested by earlier studies, is the parent-of-origin effect in the etiology of groups of CHDs. Parent-of-origin effects arise when the phenotypic impact of an allele depends on whether it is inherited from the mother or father [58]. Although several mechanisms are proposed to cause parent-of-origin effects, genomic imprinting and trans-generational effects are the two primary mechanisms that have been widely described [58–60]. Parent-of-origin effects are often associated with the imprinting principle in which an allele of a specific gene is silenced through epigenetic mechanism when inherited from one parent and expressed when inherited from the other parent [58, 60, 61]. On the other hand, transgenerational genomic effects occur due to transmitting epigenetic information from one generation to subsequent generations in the absence of direct exposure [58, 62]. In recent years, a growing body of genome-wide association studies have successfully identified parent-of-origin effects due to genomic imprinting and trans-generational inheritance in several phenotypes, including cleft lip with/without palate [63, 64], non-syndromic orofacial clefts [65], autism spectrum disorder [59, 66], attention-deficit/hyperactivity disorder [67], body mass index [68, 69], testicular germ cell tumors [70], and schizophrenia [71]. The gene expression determined by parent-of-origin effect may result in disease. Two common examples include Prader–Willi syndrome [72] and Angelman syndrome [72, 73] in which the maternal or paternal locus in the 15q11-13 region, respectively, is either silenced or removed [60]. Only a small number of imprinted loci encompassing a small portion of the human genome have been identified in the literature.

MGMT is a DNA repair enzyme that is thought to be involved in the prevention of DNA damage and oxidative stress, and the expression of *MGMT* is associated with antioxidant mechanisms [74]. Potential involvement of *MGMT* in the development of CHDs was previously suggested by our research group [75]. Due to the tendency of imprinted genes to cluster together, we examined regions within 500 Kb of imprinted genes to identify parent-of-origin effects [49]. Rs1762430 in *MGMT* has significant paternal versus maternal effects and is close to (<370bp) a known imprinted gene, *GLRX3* [76]. GLRX3, a protein in the transsulfuration pathway, is thought to be involved in cell growth, organ development, and other normal processes of growth and development [77]. Therefore, findings from our study suggest that risk of OHD associated with rs1762430 in *MGMT* may be linked to genomic imprinting of the nearby gene *GLRX3*. However, future studies of this region using gene expression profiles of parental trios would help confirm the role of imprinting in OHD risk.

Genetic variation may change the response of an individual to the exposure of environmental factors. When genetic susceptibility is high, even the minimum contribution from environmental risk factors may trigger disease development. Notable effects of environmental factors have been observed in infants with *RFC1* polymorphisms resulting in development of CHDs [78, 79]. RFC-1 is a protein involved in the folate pathway that is responsible for the transport of folate molecules from the circulation to peripheral cells and regulation of the delivery of 5-methyltetrahydrofolate from the endocytotic vesicle into the cytoplasm [80]. Several polymorphisms in the *RFC1* gene are well studied in the literature. A previous study reported that compared to infants with A80/A80 genotype, infants with G80/G80 genotype had a non-significant increased risk of conotruncal heart defects among mothers using and not using folic acid, indicating different effects of gene-environment interaction [79]. To date, no studies have identified potential associations of rs6812588 in *RFC1* and development of CHDs.

GSTA3 acts by mitigating oxidative stress in the transsulfuration pathway, which is subsequently associated with increased risk of conotruncal heart defects [53, 81]. In our study, two SNPs (rs9296695 and rs4712023) in *GSTA3* were statistically significant when comparing paternal versus maternal inheritance and OHD risk. Being located in the downstream region of the gene, these SNPs might have a regulatory role on the *GSTA3* gene. A study in the past has shown several maternal and fetal genotypes of SNPs in the glutathione transferase including GSTA3 to increase the impact of risk factors such as maternal obesity and tobacco use on the risk of CTDs [53]. Our findings suggest that *GSTA3* might play a similar role in OHD risk as that observed for conotruncal heart defects, although further studies are warranted.

While imprinted genes tend to cluster together, our study only identified imprinted genes *MGMT* and *GLRX3* together, and not *RFC1*, thus indicating a possible residual effect of *RFC1* in the occurrence of OHDs. With a limited literature on comparison of maternal and paternal genetic variants on heart defects as well as potential effects of *RFC1* [78, 79], our study findings warrant continued caution on the genomic imprinting effect of *RFC1* on OHDs.

Our search of the published English language literature in PubMed, to date, produced only one study [51] that conducted a parent-of-origin analysis for risk of CHDs as the primary aim. In that study, Nembhard et al observed that children who inherited a paternally-derived copy of the A allele for rs7818511 in the *GSR* gene, or the A allele for rs17085159 or the T allele for rs12109442 in the *GLRX* gene, were found to be at decreased risk of developing conotruncal heart defects compared to children with the maternal copy of the same allele [51]. A study by Long et al. [82] conducted an ad hoc parent-of-origin analysis to evaluate the association between maternal SNPs in folate regulated genes and the risk of left-sided heart defects and conotruncal heart defects. In that study, the primary analysis indicated that *MTR* A2756G was associated with the studied cardiac defects. Although results from parent-of-origin likelihood-ratio-test as ad hoc analysis was not statistically significant, the log-linear likelihood-ratio-test for conotruncal heart defect case triads was statistically significant. Therefore, findings from these studies further support the possibility of parent-of-origin effects in the etiology of CHDs.

Our study has several potential limitations. First, DNA was extracted from self-collected buccal cell samples; therefore, there may be an unknown level of inconsistency in the quality of the DNA samples. However, to ensure high-quality genotypes in this study, stringent quality control measures were applied by excluding SNPs with poor clustering behavior, no-call rates >10%, Mendelian error rates >5%, minor allele frequency (MAF) <5%, or significant deviation from Hardy-Weinberg equilibrium in at least one racial group. Second, we could not validate the role of imprinting on OHD due to lack of gene expression data in the case-parental trios. Third, the cases were livebirths, so the observed decreased risks may only be representative of those cases who survived. This limits the observation of a prenatal survivor effect in this study. Fourth, heterogeneity of OHDs and the associations observed in this study might be affected by the broad outcome classification. However, OHDs for both right ventricular and left ventricular groups were combined to have a sufficient sample for meaningful interpretation. This has also been done in past studies in literature to have adequate genotyped sample for meaningful interpretations [54, 75, 83]. Fifth, our study did not include information on paternal smoking, alcohol consumption and occupation because of limited data availability and our major focus on genotyped information. A recent systematic review suggested advanced paternal age, smoking, alcohol consumption and specific occupations were associated with an increased risk of CHDs [34]. While we were able to assess paternal employment status, further studies assessing specific paternal risk factors, including smoking, alcohol consumption and occupations, in association with genetic variants would help determine risk specific to OHDs. Despite these overall limitations, our study possesses several strengths. First, in this large population-based study, our study population consisted of multiple racial/ethnic groups. Second, all the OHD cases were confirmed by pediatric cardiologists and a standard procedure for the OHD classification was used across participating study centers. Finally, in addition to exploring effects of inherited genotypes, the usage of log-linear modelling allowed us to also examine prenatal effects of maternal genotype and parent-of-origin (imprinting) effects. Furthermore log-linear models bear an advatange over other models as they can be extended to any number of alleles or loci, or any number of risk factors.

In conclusion, we observed that paternal genetic variants in certain folate and transsulfuration pathway genes contributed to a lower risk of OHDs occurence compared to maternal variants. Future studies with larger sample size and multi-omics data are indicated to validate our findings to gain additional confidence.

## Methods and materials

### Ethics statement

The Institutional Review Boards (IRB) at each of the following collaborative centers of the National Birth Defects Prevention Study (NBDPS) provided approval: University of Arkansas for Medical Sciences, California Birth Defects Monitoring Program, University of Iowa, Massachusetts Department of Public Health, New Jersey Department of Health, State of New York Department of Health, University of North Carolina at Chapel Hill, Texas Department of State Health Services, and University of Utah. All study participants (including parents of minors) provided written or verbal informed consent. For the telephone interview, each participant provided a verbal consent (permission) to use their answers in the study to understand the causes of birth defects. For the DNA samples, participants also provided a signed written consent through a form that was provided with the collection kit through mail, that also explained the study risks and benefits. The Centers for Disease Control and Prevention Institutional Review Board (IRB), along with the IRBs for each participating center, have approved the NBDPS [84, 85].

### National Birth Defects Prevention Study

The NBDPS is one of the largest population-based case-control studies of birth defects conducted in the United States. and provides a unique opportunity to examine genetic, environmental, and behavior factors associated with the occurrence of major non-syndromic birth defects. Methods of the NBDPS have been previously described [84, 85], but in brief, families of case and control infants were identified from population-based birth defects surveillance systems in 10 states: Arkansas, California, Georgia, Iowa, Massachusetts, New Jersey, New York, North Carolina, Texas, and Utah [84, 85]. The study enrolled approximately 44,000 women who were non-Hispanic (NH) white, NH-black, Hispanic, and of other races with estimated dates of delivery from October 1, 1997 through December 31, 2011. The case-only study we conducted included a subset of women with estimated dates of delivery between October 1997 and August 2008, for whom DNA specimens were available from themselves, their infants, and the infant’s father [84, 85].

### Ascertainment of obstructive heart defects

In the NBDPS, a range of non-syndromic CHD case infants were identified following diagnostic procedures, including echocardiogram, surgical reports, cardiac catherization, or autopsy. Each diagnostic procedure result was further reviewed by a pediatric cardiologist to ensure consistent diagnoses across the study. A classification system was developed specifically for the NBDPS and included cardiac phenotype, cardiac complexity, and extra-cardiac anomalies. Our study included left and right-sided OHDs. These were grouped together since we had limited sample size for individual phenotypes due to the overall smaller sample of genotyped CHDs. The left-sided OHDs were comprised of hypoplastic left heart syndrome, interrupted aortic arch A, coarctation of the aorta, and aortic stenosis; the right-sided OHDs were comprised of pulmonary valve stenosis, tricuspid atresia, and Ebstein anomaly.[86]

### Maternal interview

After informed consent, women completed a 1-hour computer-assisted telephone interview in either English or Spanish from 6 weeks to 2 years after their estimated date of delivery. Overall interview participation rate was 67% among case women and 65% among control women. Mean number of weeks of gestation was 37.1 for cases and 38.7 for controls. Interviewers obtained information on maternal demographic characteristics and other risk factors (e.g., maternal health, pregnancy, diet/substance use, home/work, family demographics, and medication use) both before and during pregnancy. Information on the father of the infant was also collected during the interview.

### DNA sample collection

Upon completing the telephone interview, families were mailed buccal cell collection kits. These kits were used to collect specimens for maternal, infant, and paternal DNA. Details on methods for DNA extraction, purification from buccal cell swabs and storage were described previously [84, 87]. In this study, a customized panel, including 1536 SNPs from 62 genes that were part of one-carbon metabolism (i.e., folate, homocysteine and glutathione/transsulfuration pathways), was used for genotyping on Illumina’s GoldenGate platform [53, 88]. 10–15 ng of genomic DNA was used for whole genome amplification (WGA) using GenomePl WGA kit [89]. The WGA product was quantified using TaqMan RNase Reagent Kit [89]. Genotype clustering and calling was conducted using a previously developed and tested genotype calling algorithm, SNPMClust, that was developed in-house at the Arkansas Center for Birth Defects Research and Prevention [90]. To ensure high-quality genotypes, we applied stringent quality control measures and excluded SNPs with poor clustering behavior, no-call rates >10%, greater Mendelian error rates >5%, MAF <5%, or significant deviation from Hardy-Weinberg equilibrium in at least one racial group. Given the apparent lack of genome-wide data, racial group was considered as a proxy for genetic ancestry. For the current study specific to OHDs, the final dataset included 1203 individuals from 569 case families, each with 877 SNPs. Of the 569 case families, 220 (38.7%) were full trios.

### Statistical methods

Summary statistics were expressed as means (standard deviation) for continuous variables, and counts (percentage) for categorical variables. To investigate the parent-of-origin effect in this case-only study, a log-linear model was fitted for the counts of each SNP as a function of mating types, maternal genetic effect, and imprinting parameter [30]. Based on the log-linear model for counts and assuming a Poisson distribution, the imprinting effect was estimated as the relative risk of an OHD in a child who inherited a paternally-derived copy of the minor allele compared to a child who inherits a copy of the minor allele from the mother. Bonferroni correction was used to adjust for multiple testing. Statistical significance level was set at *p* < 5.70 x 10^−5^ based on log likelihood statistics. Data were analyzed using statistical software SAS 9.4 (SAS Institute Inc., Cary, NC) for computing descriptive statistics and PREMIM/EMIM for fitting imprinting models. EMIM uses the genotype data to perform statistical analysis, while PREMIM allows the extraction of genotype data from pedigree data files. This technique allows the estimation of complex genetic effects such as parent-of-origin (imprinting) effects [91].

## References

[1] Bernier P-L, Stefanescu A, Samoukovic G, Tchervenkov CI, editors. The challenge of congenital heart disease worldwide: epidemiologic and demographic facts. Seminars in Thoracic and Cardiovascular Surgery: Pediatric Cardiac Surgery Annual; 2010: Elsevier. 10.1053/j.pcsu.2010.02.005 20307858

[2] van der Linde D, Konings EEM, Slager MA, Witsenburg M, Helbing WA, Takkenberg JJ, et al. Birth prevalence of congenital heart disease worldwide: A systematic review and meta-analysis. Journal of the American College of Cardiology. 2011;58(21):2241–7. 10.1016/j.jacc.2011.08.025 22078432

[3] Verheugt CL, Uiterwaal CS, Grobbee DE, Mulder BJ. Long-term prognosis of congenital heart defects: a systematic review. International Journal of Cardiology. 2008;131(1):25–32. 10.1016/j.ijcard.2008.06.023 18687485

[4] Gilboa SM, Salemi JL, Nembhard WN, Fixler DE, Correa A. Mortality resulting from congenital heart disease among children and adults in the United States, 1999 to 2006. Circulation. 2010;122(22):2254–63. 10.1161/CIRCULATIONAHA.110.947002 21098447 PMC4911018

[5] Hobbs CA, MacLeod SL, James S, Cleves MA. Congenital heart defects and maternal genetic, metabolic, and lifestyle factors. Birth Defects Research Part A: Clinical and Molecular Teratology. 2011;91(4):195–203. 10.1002/bdra.20784 21384532

[6] Jenkins KJ, Correa A, Feinstein JA, Botto L, Britt AE, Daniels SR, et al. Noninherited risk factors and congenital cardiovascular defects: current knowledge: a scientific statement from the American Heart Association Council on Cardiovascular Disease in the Young: endorsed by the American Academy of Pediatrics. Circulation. 2007;115(23):2995–3014. 10.1161/CIRCULATIONAHA.106.183216 17519397

[7] Matthews V, Schuster B, Schütze S, Bussmeyer I, Ludwig A, Hundhausen C, et al. Cellular cholesterol depletion triggers shedding of the human interleukin-6 receptor by ADAM10 and ADAM17 (TACE). Journal of Biological Chemistry. 2003;278(40):38829–39. 10.1074/jbc.M210584200 12832423

[8] Tinker SC, Gilboa SM, Moore CA, Waller DK, Simeone RM, Kim SY, et al. Specific birth defects in pregnancies of women with diabetes: National Birth Defects Prevention Study, 1997–2011. American Journal of Obstetrics and Gynecology. 2020;222(2):176. e1-. e11. 10.1016/j.ajog.2019.08.028 31454511 PMC7186569

[9] Persson M, Razaz N, Bonamy A-KE, Villamor E, Cnattingius S. Maternal overweight and obesity and risk of congenital heart defects. Journal of the American College of Cardiology. 2019;73(1):44–53. 10.1016/j.jacc.2018.10.050 30621950

[10] Beal MA, Yauk CL, Marchetti F. From sperm to offspring: Assessing the heritable genetic consequences of paternal smoking and potential public health impacts. Mutation Research/Reviews in Mutation Research. 2017;773:26–50. 10.1016/j.mrrev.2017.04.001 28927533

[11] Deng K, Liu Z, Lin Y, Mu D, Chen X, Li J, et al. Periconceptional paternal smoking and the risk of congenital heart defects: a case-control study. Birth Defects Research Part A: Clinical and Molecular Teratology. 2013;97(4):210–6. 10.1002/bdra.23128 23554276

[12] Hobbs CA, James S, Jernigan S, Melnyk S, Lu Y, Malik S, et al. Congenital heart defects, maternal homocysteine, smoking, and the 677 C> T polymorphism in the methylenetetrahydroflate reductase gene: Evaluating gene-environment interactions. American Journal of Obstetrics and Gynecology. 2006;194(1):218–24. 10.1016/j.ajog.2005.06.016 16389035

[13] Källén K. Maternal smoking and congenital heart defects. European Journal of Epidemiology. 1999;15(8):731–7. 10.1023/a:1007671631188 10555617

[14] Karatza AA, Giannakopoulos I, Dassios TG, Belavgenis G, Mantagos SP, Varvarigou AA. Periconceptional tobacco smoking and Xisolated congenital heart defects in the neonatal period. International Journal of Cardiology. 2011;148(3):295–9. 10.1016/j.ijcard.2009.11.008 19951824

[15] Malik S, Cleves MA, Honein MA, Romitti PA, Botto LD, Yang S, et al. Maternal smoking and congenital heart defects. Pediatrics. 2008;121(4):e810–e6. 10.1542/peds.2007-1519 18381510

[16] Mao B, Qiu J, Zhao N, Shao Y, Dai W, He X, et al. Maternal folic acid supplementation and dietary folate intake and congenital heart defects. PloS One. 2017;12(11). 10.1371/journal.pone.0187996 29145433 PMC5690601

[17] Verkleij-Hagoort AC, Verlinde M, Ursem NTC, Lindemans J, Helbing WA, Ottenkamp J, et al. Maternal hyperhomocysteinaemia is a risk factor for congenital heart disease. BJOG: An International Journal of Obstetrics & Gynaecology. 2006;113(12):1412–8. 10.1111/j.1471-0528.2006.01109.x 17081182

[18] Caton AR, Bell EM, Druschel CM, Werler MM, Lin AE, Browne ML, et al. Antihypertensive medication use during pregnancy and the risk of cardiovascular malformations. Hypertension. 2009;54(1):63–70. 10.1161/HYPERTENSIONAHA.109.129098 19433779 PMC4913772

[19] Chowdhury S, Hobbs CA, MacLeod SL, Cleves MA, Melnyk S, James SJ, et al. Associations between maternal genotypes and metabolites implicated in congenital heart defects. Molecular Genetics and Metabolism. 2012;107(3):596–604. 10.1016/j.ymgme.2012.09.022 23059056 PMC3523122

[20] Hobbs CA, Cleves MA, Karim MA, Zhao W, MacLeod SL. Maternal folate-related gene environment interactions and congenital heart defects. Obstetrics and Gynecology. 2010;116(2 Pt 1):316. 10.1097/AOG.0b013e3181e80979 20664391 PMC3027124

[21] Shaw GM, Nelson V, Moore CA. Prepregnancy body mass index and risk of multiple congenital anomalies. American Journal of Medical Genetics. 2002;107(3):253–5. 10.1002/ajmg.10164 11807910

[22] Waller DK, Mills JL, Simpson JL, Cunningham GC, Conley MR, Lassman MR, et al. Are obese women at higher risk for producing malformed offspring? American Journal of Obstetrics and Gynecology. 1994;170(2):541–8. 10.1016/s0002-9378(94)70224-1 8116710

[23] Botto LD, Mulinare J, Erickson JD. Do multivitamin or folic acid supplements reduce the risk for congenital heart defects? Evidence and gaps. American Journal of Medical Genetics Part A. 2003;121(2):95–101. 10.1002/ajmg.a.20132 12910485

[24] Czeizel AE. Periconceptional folic acid containing multivitamin supplementation. European Journal of Obstetrics & Gynecology and Reproductive Biology. 1998;78(2):151–61. 10.1016/s0301-2115(98)00061-x 9622312

[25] Hobbs CA, Cleves MA, Melnyk S, Zhao W, James SJ. Congenital heart defects and abnormal maternal biomarkers of methionine and homocysteine metabolism. The American Journal of Clinical Nutrition. 2005;81(1):147–53. 10.1093/ajcn/81.1.147 15640474

[26] Maurano MT, Humbert R, Rynes E, Thurman RE, Haugen E, Wang H, et al. Systematic localization of common disease-associated variation in regulatory DNA. Science. 2012;337(6099):1190–5. 10.1126/science.1222794 22955828 PMC3771521

[27] Patel SS, Burns TL, Botto LD, Riehle-Colarusso TJ, Lin AE, Shaw GM, et al. Analysis of selected maternal exposures and non-syndromic atrioventricular septal defects in the National Birth Defects Prevention Study, 1997–2005. American Journal of Medical Genetics Part A. 2012;158(10):2447–55. 10.1002/ajmg.a.35555 PMC4462202 22903798

[28] Scanlon KS, Ferencz C, Loffredo CA, Wilson PD, Correa-Villaseñor A, Khoury MJ, et al. Preconceptional folate intake and malformations of the cardiac outflow tract. Epidemiology. 1998:95–8. 9430276

[29] Wenstrom KD, Johanning GL, Johnston KE, DuBard M. Association of the C677T methylenetetrahydrofolate reductase mutation and elevated homocysteine levels with congenital cardiac malformations. American Journal of Obstetrics and Gynecology. 2001;184(5):806–17. 10.1067/mob.2001.113845 11303187

[30] Weinberg CR. Methods for detection of parent-of-origin effects in genetic studies of case-parents triads. The American Journal of Human Genetics. 1999;65(1):229–35. 10.1086/302466 10364536 PMC1378094

[31] Li X, Li S, Mu D, Liu Z, Li Y, Lin Y, et al. The association between periconceptional folic acid supplementation and congenital heart defects: a case–control study in China. Preventive Medicine. 2013;56(6):385–9. 10.1016/j.ypmed.2013.02.019 23480969

[32] van Beynum IM, Kapusta L, Bakker MK, den Heijer M, Blom HJ, de Walle HE. Protective effect of periconceptional folic acid supplements on the risk of congenital heart defects: a registry-based case–control study in the northern Netherlands. European Heart Journal. 2010;31(4):464–71. 10.1093/eurheartj/ehp479 19952004

[33] Ngwezi DP, Hornberger LK, Vargas AO. Environmental pollution and the development of congenital heart disease: A scoping review. Advances in Pediatric Research. 2018;5:17.

[34] Peng J, Meng Z, Zhou S, Zhou Y, Wu Y, Wang Q, et al. The non-genetic paternal factors for congenital heart defects: A systematic review and meta-analysis. Clinical Cardiology. 2019;42(7):684–91. 10.1002/clc.23194 31073996 PMC6605632

[35] Olshan AF, Schnitzer PG, Baird PA. Paternal age and the risk of congenital heart defects. Teratology. 1994;50(1):80–4. 10.1002/tera.1420500111 7974258

[36] Green RF, Devine O, Crider KS, Olney RS, Archer N, Olshan AF, et al. Association of paternal age and risk for major congenital anomalies from the National Birth Defects Prevention Study, 1997 to 2004. Annals of Epidemiology. 2010;20(3):241–9. 10.1016/j.annepidem.2009.10.009 20056435 PMC2824069

[37] Lian ZH, Zack MM, Erickson JD. Paternal age and the occurrence of birth defects. American Journal of Human Genetics. 1986;39(5):648. 3788977 PMC1684057

[38] Ou Y, Mai J, Zhuang J, Liu X, Wu Y, Gao X, et al. Risk factors of different congenital heart defects in Guangdong, China. Pediatric Research. 2016;79(4):549–58. 10.1038/pr.2015.264 26679154

[39] Su XJ, Yuan W, Huang GY, Olsen J, Li J. Paternal age and offspring congenital heart defects: a national cohort study. PLoS One. 2015;10(3). 10.1371/journal.pone.0121030 25806788 PMC4373953

[40] Zhan SY, Lian ZH, Zheng DZ, Gao L. Effect of fathers’ age and birth order on occurrence of congenital heart disease. Journal of Epidemiology & Community Health. 1991;45(4):299–301. 10.1136/jech.45.4.299 1795151 PMC1059465

[41] Cedergren MI, Selbing AJ, Källén BA. Risk factors for cardiovascular malformation—a study based on prospectively collected data. Scandinavian Journal of Work, Environment & Health. 2002:12–7. 10.5271/sjweh.641 11873776

[42] Cresci M, Foffa I, Ait-Ali L, Pulignani S, Gianicolo EAL, Botto N, et al. Maternal and paternal environmental risk factors, metabolizing GSTM1 and GSTT1 polymorphisms, and congenital heart disease. The American Journal of Cardiology. 2011;108(11):1625–31. 10.1016/j.amjcard.2011.07.022 21890078

[43] Wang C, Zhan Y, Wang F, Li H, Xie L, Liu B, et al. Parental occupational exposures to endocrine disruptors and the risk of simple isolated congenital heart defects. Pediatric Cardiology. 2015;36(5):1024–37. 10.1007/s00246-015-1116-6 25628158

[44] Linschooten JO, Verhofstad N, Gutzkow K, Olsen A-K, Yauk C, Oligschläger Y, et al. Paternal lifestyle as a potential source of germline mutations transmitted to offspring. The FASEB Journal. 2013;27(7):2873–9. 10.1096/fj.13-227694 23538710 PMC3688758

[45] Marchetti F, Rowan-Carroll A, Williams A, Polyzos A, Berndt-Weis ML, Yauk CL. Sidestream tobacco smoke is a male germ cell mutagen. Proceedings of the National Academy of Sciences. 2011;108(31):12811–4. 10.1073/pnas.1106896108 21768363 PMC3150936

[46] Sharma R, Agarwal A, Rohra VK, Assidi M, Abu-Elmagd M, Turki RF. Effects of increased paternal age on sperm quality, reproductive outcome and associated epigenetic risks to offspring. Reproductive Biology and Endocrinology. 2015;13(1):35. 25928123 10.1186/s12958-015-0028-x PMC4455614

[47] Wyrobek AJ, Eskenazi B, Young S, Arnheim N, Tiemann-Boege I, Jabs EW, et al. Advancing age has differential effects on DNA damage, chromatin integrity, gene mutations, and aneuploidies in sperm. Proceedings of the National Academy of Sciences. 2006;103(25):9601–6. 10.1073/pnas.0506468103 PMC1480453 16766665

[48] Yauk CL, Berndt ML, Williams A, Rowan-Carroll A, Douglas GR, Stämpfli MR. Mainstream tobacco smoke causes paternal germ-line DNA mutation. Cancer Research. 2007;67(11):5103–6. 10.1158/0008-5472.CAN-07-0279 17545587

[49] Kong A, Frigge ML, Masson G, Besenbacher S, Sulem P, Magnusson G, et al. Rate of de novo mutations and the importance of father’s age to disease risk. Nature. 2012;488(7412):471–5. 10.1038/nature11396 22914163 PMC3548427

[50] Zhu H, Yang W, Lu W, Etheredge AJ, Lammer EJ, Finnell RH, et al. Gene variants in the folate-mediated one-carbon metabolism (FOCM) pathway as risk factors for conotruncal heart defects. American Journal of Medical Genetics Part A. 2012;158(5):1124–34. 10.1002/ajmg.a.35313 22495907 PMC3331895

[51] Nembhard WN, Tang X, Li J, MacLeod SL, Levy J, Schaefer GB, et al. A parent-of-origin analysis of paternal genetic variants and increased risk of conotruncal heart defects. American Journal of Medical Genetics Part A. 2018;176(3):609–17. 10.1002/ajmg.a.38611 29399948 PMC5881110

[52] Hobbs CA, Cleves MA, Zhao W, Melnyk S, James SJ. Congenital heart defects and maternal biomarkers of oxidative stress. The American Journal of Clinical Nutrition. 2005;82(3):598–604. 10.1093/ajcn.82.3.598 16155273

[53] Tang X, Nick TG, Cleves MA, Erickson SW, Li M, Li J, et al. Maternal obesity and tobacco use modify the impact of genetic variants on the occurrence of conotruncal heart defects. PloS One. 2014;9(10). 10.1371/journal.pone.0108903 25275547 PMC4183535

[54] Tang X, Cleves MA, Nick TG, Li M, MacLeod SL, Erickson SW, et al. Obstructive heart defects associated with candidate genes, maternal obesity, and folic acid supplementation. American Journal of Medical Genetics Part A. 2015;167(6):1231–42. 10.1002/ajmg.a.36867 25846410 PMC4675451

[55] Botto LD, Olney RS, Erickson JD, editors. Vitamin supplements and the risk for congenital anomalies other than neural tube defects. American Journal of Medical Genetics Part C: Seminars in Medical Genetics; 2004: Wiley Online Library. 10.1002/ajmg.c.30004 14755429

[56] Kuehl KS, Loffredo CA. Genetic and environmental influences on malformations of the cardiac outflow tract. Expert Review of Cardiovascular Therapy. 2005;3(6):1125–30. 10.1586/14779072.3.6.1125 16293002

[57] Hobbs CA, James S, Parsian A, Krakowiak PA, Jernigan S, Greenhaw JJ, et al. Congenital heart defects and genetic variants in the methylenetetrahydroflate reductase gene. Journal of Medical Genetics. 2006;43(2):162–6. 10.1136/jmg.2005.032656 15951337 PMC2564637

[58] Lawson HA, Cheverud JM, Wolf JB. Genomic imprinting and parent-of-origin effects on complex traits. Nature Reviews Genetics. 2013;14(9):609–17. 10.1038/nrg3543 23917626 PMC3926806

[59] Connolly S, Heron EA. Review of statistical methodologies for the detection of parent-of-origin effects in family trio genome-wide association data with binary disease traits. Briefings in Bioinformatics. 2015;16(3):429–48. 10.1093/bib/bbu017 24903222

[60] Elhamamsy AR. Role of DNA methylation in imprinting disorders: an updated review. Journal of Assisted Reproduction and Genetics. 2017;34(5):549–62. 10.1007/s10815-017-0895-5 28281142 PMC5427654

[61] Rampersaud E, Mitchell BD, Naj AC, Pollin TI. Investigating parent of origin effects in studies of type 2 diabetes and obesity. Current Diabetes Reviews. 2008;4(4):329–39. 10.2174/157339908786241179 18991601 PMC2896493

[62] Nelson VR, Nadeau JH. Transgenerational genetic effects. Epigenomics. 2010;2(6):797–806. 10.2217/epi.10.57 22122083 PMC3720237

[63] Sull JW, Liang K-Y, Hetmanski JB, Wu T, Fallin MD, Ingersoll RG, et al. Evidence that TGFA influences risk to cleft lip with/without cleft palate through unconventional genetic mechanisms. Human Genetics. 2009;126(3):385–94. 10.1007/s00439-009-0680-3 19444471 PMC2901599

[64] Duan S-J, Huang N, Zhang B-H, Shi J-Y, He S, Ma J, et al. New insights from GWAS for the cleft palate among han Chinese population. Medicina Oral Patologia Oral y Cirugia Bucal. 2017;22(2):e219. 10.4317/medoral.21439 28160584 PMC5359705

[65] Garg P, Ludwig KU, Böhmer AC, Rubini M, Steegers-Theunissen R, Mossey PA, et al. Genome-wide analysis of parent-of-origin effects in non-syndromic orofacial clefts. European Journal of Human Genetics. 2014;22(6):822–30. 10.1038/ejhg.2013.235 24169523 PMC4023210

[66] Connolly S, Anney R, Gallagher L, Heron EA. A genome-wide investigation into parent-of-origin effects in autism spectrum disorder identifies previously associated genes including SHANK3. European Journal of Human Genetics. 2017;25(2):234–9. 10.1038/ejhg.2016.153 27876814 PMC5255953

[67] Wang K-S, Liu X, Zhang Q, Aragam N, Pan Y. Parent-of-origin effects of FAS and PDLIM1 in attention-deficit/hyperactivity disorder. Journal of Psychiatry & Neuroscience:. 2012;37(1):46. 10.1503/jpn.100173 21651830 PMC3244498

[68] Hoggart CJ, Venturini G, Mangino M, Gomez F, Ascari G, Zhao JH, et al. Novel approach identifies SNPs in SLC2A10 and KCNK9 with evidence for parent-of-origin effect on body mass index. PLoS Genetics. 2014;10(7). 10.1371/journal.pgen.1004508 25078964 PMC4117451

[69] Liu X, Hinney A, Scholz M, Scherag A, Tönjes A, Stumvoll M, et al. Indications for potential parent-of-origin effects within the FTO gene. PloS One. 2015;10(3). 10.1371/journal.pone.0119206 25793382 PMC4368796

[70] Karlsson R, Andreassen KE, Kristiansen W, Aschim EL, Bremnes RM, Dahl O, et al. Investigation of six testicular germ cell tumor susceptibility genes suggests a parent-of-origin effect in SPRY4. Human Molecular Genetics. 2013;22(16):3373–80. 10.1093/hmg/ddt188 23640991

[71] Palmer CGS, Hsieh H-J, Reed EF, Lonnqvist J, Peltonen L, Woodward JA, et al. HLA-B maternal-fetal genotype matching increases risk of schizophrenia. The American Journal of Human Genetics. 2006;79(4):710–5. 10.1086/507829 16960807 PMC1592576

[72] Shemer R, Hershko AY, Perk J, Mostoslavsky R, Tsuberi B-z, Cedar H, et al. The imprinting box of the Prader-Willi/Angelman syndrome domain. Nature Genetics. 2000;26(4):440–3. 10.1038/82571 11101841

[73] Buiting K, Williams C, Horsthemke B. Angelman syndrome—insights into a rare neurogenetic disorder. Nature Reviews Neurology. 2016;12(10):584. 10.1038/nrneurol.2016.133 27615419

[74] Niture SK, Velu CS, Smith QR, Bhat GJ, Srivenugopal KS. Increased expression of the MGMT repair protein mediated by cysteine prodrugs and chemopreventative natural products in human lymphocytes and tumor cell lines. Carcinogenesis. 2007;28(2):378–89. 10.1093/carcin/bgl155 16950796

[75] Li M, Cleves MA, Mallick H, Erickson SW, Tang X, Nick TG, et al. A genetic association study detects haplotypes associated with obstructive heart defects. Human Genetics. 2014;133(9):1127–38. 10.1007/s00439-014-1453-1 24894164 PMC4313870

[76] Partida GC, Laurin C, Ring SM, Gaunt TR, Relton CL, Smith GD, et al. Imprinted loci may be more widespread in humans than previously appreciated and enable limited assignment of parental allelic transmissions in unrelated individuals. BioRxiv. 2017:161471.

[77] Cheng NH, Zhang W, Chen WQ, Jin J, Cui X, Butte NF, et al. A mammalian monothiol glutaredoxin, Grx3, is critical for cell cycle progression during embryogenesis. The FEBS Journal. 2011;278(14):2525–39. 10.1111/j.1742-4658.2011.08178.x 21575136 PMC4038268

[78] Hobbs CA, Cleves MA, MacLeod SL, Erickson SW, Tang X, Li J, et al. Conotruncal heart defects and common variants in maternal and fetal genes in folate, homocysteine, and transsulfuration pathways. Birth Defects Research Part A: Clinical and Molecular Teratology. 2014;100(2):116–26. 10.1002/bdra.23225 24535845 PMC4118819

[79] Shaw GM, Zhu H, Lammer EJ, Yang W, Finnell RH. Genetic variation of infant reduced folate carrier (A80G) and risk of orofacial and conotruncal heart defects. American Journal of Epidemiology. 2003;158(8):747–52. 10.1093/aje/kwg189 14561664

[80] Chango A, Emery-Fillon N, de Courcy GP, Lambert D, Pfister M, Rosenblatt DS, et al. A polymorphism (80G-> A) in the reduced folate carrier gene and its associations with folate status and homocysteinemia. Molecular Genetics and Metabolism. 2000;70(4):310–5. 10.1006/mgme.2000.3034 10993718

[81] Edwards JJ, Gelb BD. Genetics of congenital heart disease. Current Opinion in Cardiology. 2016;31(3):235. 10.1097/HCO.0000000000000274 26872209 PMC4868504

[82] Long J, Lupo PJ, Goldmuntz E, Mitchell LE. Evaluation of heterogeneity in the association between congenital heart defects and variants of folate metabolism genes: Conotruncal and left-sided cardiac defects. Birth Defects Research Part A: Clinical and Molecular Teratology. 2011;91(10):879–84. 10.1002/bdra.22849 21987465 PMC3257803

[83] Tang X, Eberhart JK, Cleves MA, Li J, Li M, MacLeod S, et al. PDGFRA gene, maternal binge drinking and obstructive heart defects. Scientific reports. 2018;8(1):1–7. 10.1038/s41598-017-17765-5 30038270 PMC6056529

[84] Reefhuis J, Gilboa SM, Anderka M, Browne ML, Feldkamp ML, Hobbs CA, et al. The national birth defects prevention study: a review of the methods. Birth Defects Research Part A: Clinical and Molecular Teratology. 2015;103(8):656–69. 10.1002/bdra.23384 26033852 PMC4558899

[85] Yoon PW, Rasmussen SA, Lynberg MC, Moore CA, Anderka M, Carmichael SL, et al. The National Birth Defects Prevention Study. Public Health Reports. 2001;116(Suppl 1):32. 10.1093/phr/116.S1.32 11889273 PMC1913684

[86] Botto LD, Lin AE, Riehle-Colarusso T, Malik S, Correa A. Seeking causes: classifying and evaluating congenital heart defects in etiologic studies. Birth Defects Research Part A: Clinical and Molecular Teratology. 2007;79(10):714–27. 10.1002/bdra.20403 17729292

[87] Gallagher ML, Sturchio C, Smith A, Koontz D, Jenkins MM, Honein MA, et al. Evaluation of mailed pediatric buccal cytobrushes for use in a case-control study of birth defects. Birth Defects Research Part A: Clinical and Molecular Teratology. 2011;91(7):642–8. 10.1002/bdra.20829 21630425

[88] Fan J-B, Chee MS, Gunderson KL. Highly parallel genomic assays. Nature Reviews Genetics. 2006;7(8):632–44. 10.1038/nrg1901 16847463

[89] Ács N, Bánhidy F, Puhó EH, Czeizel AE. Possible association between acute pelvic inflammatory disease in pregnant women and congenital abnormalities in their offspring: A population-based case-control study. Birth Defects Research Part A: Clinical and Molecular Teratology. 2008;82(8):563–70. 10.1002/bdra.20480 18553461

[90] Erickson SW, Callaway JC. SNPMClust: Bivariate Gaussian Genotype Clustering and Calling for Illumina Microarrays. Journal of Statistical Software. 2016;10.

[91] Howey R, Cordell HJ. PREMIM and EMIM: tools for estimation of maternal, imprinting and interaction effects using multinomial modelling. BMC Bioinformatics. 2012;13(1):149. 10.1186/1471-2105-13-149 22738121 PMC3464602

